# Case of Portal Phlebitis

**Published:** 1853-04

**Authors:** 


*Case of Portal Phlebitis.* Reported by C. E. ISAACS, M. D.

On the 4th of January, 1853, I was requested by Dr. Blakeman, to make a post-mortem examination of the body of a man aged 25, who, for some five or six weeks previously, had complained of "dyspeptic symptoms," and occasional attacks of colic. On the first of December, while working in his store, he was seized with very severe and griping pains in his bowels; these were relieved, at the time, by brandy. He took, the next day, some of Lee's pills, followed by castor oil, which, however, did not operate until three days afterward. Dr. Blakeman was called on the 8th, and found him complaining of some slight derangement of the stomach, want of appetite, etc., etc. Calomel and rhubarb were given, and operated easily and freely, with much relief. The bowels remained unusually open for several days after. On the 12th, the patient felt so well that he went out to visit some of his friends, but the pain in his bowels returned, and he sent for his physician. He found him free from his pains, which he described as having been very severe and spasmodic, with a feeling of constriction around the abdomen. These symptoms continued, with intermissions, for several days. There was no swelling, nor distension, nor any tenderness on pressure, although repeated examinations were made with reference to these points. He could not lie on his right side, and severe dragging pain was felt whenever he attempted to do so. It should be mentioned, that when first taken sick, he experienced a slight *chill*, that he had four chills during the progress of his disease, and that they were about one week apart. The first was not followed by sweating, but the others were so. The last *chill* was on the 25th, lasted one hour, and was followed by the most profuse sweating. Previously to this, the patient had sat up, and walked about; but after this occurrence, he was entirely confined to his bed. He could sleep only by taking large doses of morphia, to relieve pain. The pulse, during the course of the disease, was about 80, but after the last chill, rose to 120, and upward, and so continued until his death.

With the griping pains, there was occasionally nausea, and sometimes vomiting, during the whole continuance of the disease; but, generally, the appetite was tolerably good. The skin was usually moist and cool. He had, at times during his illness, passed bloody urine; occasionally it was loaded with lithic acid, would suddenly become clear, and again bloody, etc., etc. Pyemia was diagnosed by Drs. Blakeman and Post, some days previously to death.

*Post-mortem.* Present, Dr. Blakeman, Professor A. C. Post, and Dr. Eastman, of Owego. The omentum was very thin, and spread out over the surface of the intestines. On raising it up, a large drop of purulent matter was perceived, which induced me to make the examination with great care and caution; and it was at length ascertained that the upper part of the ilium had passed through a rupture, or opening, in the omentum; and had been *partially* strangulated by the latter. Above the constriction, the jejunum was very much distended, being nearly as large as the colon, while below, the ilium was of the usual size; abscesses existed throughout the mesentery, and purulent matter in its veins. On removing the liver, and laying open the *vena porta*, this vein was found to contain purulent matter. A mass of coagulable lymph and partially discolored blood was tightly adherent to the internal serous lining of that vessel. On cutting across the liver, in various directions, pus issued from the cut orifices of the branches of the portal vein. The hepatic veins were healthy. The spleen, where cut across, showed *various* points of purulent matter throughout its substance. The matter from the portal veins, and also that from the spleen, was examined microscopically by Dr. Clark, and exhibited very clearly globules of pus. Above the constriction, jejunum was more vascular than natural, and there were some flakes of coagulable lymph on its outer surface. The kidneys were not remarkable, but on pressing on the cut surface of the pyramidal portions, semi-purulent matter could be pressed out. Blood was extravasated in small spots under the mucous membrane of the bladder. The other organs were in a healthy condition. The constriction had been partial,—not enough to entirely interrupt the passage of substances through the small intestines, but sufficient to cause obstruction to the intestinal circulation, and consequent inflammation of the portal veins with abscess of the mesentery.—*N. Y. Med. Times.*

